# Integrative multi-omics analysis identifies endocrine-disrupting chemical-related molecular mechanisms in migraine

**DOI:** 10.1186/s10194-026-02412-0

**Published:** 2026-06-08

**Authors:** Yinfan Ren, Ziqi Xu, Zifei Xu, Guifang Zhang, Huanjie Huang, Chuhuai Wang, Xiao-Qian Hu

**Affiliations:** 1 Department of Rehabilitation Medicine, The First Affiliated Hospital, Sun Yat -Sen University, Guangzhou, 510080 China; 2 Clinical Medical College, Southwest Medical University, Luzhou, 646000 China; 3 Guangdong Engineering and Technology Research Centre of Rehabilitation Medicine and Translation, Guangzhou, China; 4 Guangdong Provincial Clinical Research Center for Rehabilitation Medicine, Guangzhou, China

**Keywords:** Migraine, Endocrine-disrupting chemicals (EDC), Summary data-based mendelian randomization (SMR), Multi-omics, Gene-environment interaction

## Abstract

**Background:**

Migraine is a highly prevalent, heritable neurological disorder with a marked female predominance, indicating substantial hormonal contributions to disease pathophysiology. Endocrine-disrupting chemicals (EDCs) are ubiquitous environmental contaminants that interfere with hormonal signaling and have been linked to multiple diseases, yet their molecular contribution to migraine remains unclear. This study aims to identify EDC-related genes contributing to migraine risk and explore underlying mechanisms using an integrative multi-omics and causal inference framework.

**Methods:**

We curated 181 EDCs and identified 1,116 EDC-related genes through chemical-gene interaction network analysis, followed by integration of multi-omics quantitative trait loci datasets (eQTL, sQTL, mQTL, and pQTL) to derive genetic instruments. Summary data-based Mendelian randomization (SMR) was performed to assess the effects of genetically predicted molecular traits on migraine risk using genome-wide association study (GWAS) data from the OpenGWAS and GWASCatalog. HEIDI testing and Bayesian colocalization analyses were conducted to distinguish pleiotropy from linkage and to prioritize genes with shared causal variants, followed by transcriptome-wide association study (TWAS) for validation. Multi-omics integration and differential gene expression analysis were used to characterize regulatory mechanisms. Finally, molecular docking and molecular dynamics simulations were performed to evaluate interactions between prioritized EDCs and molecular targets implicated in migraine pathophysiology.

**Results:**

Eight EDC-related genes were identified as significantly associated with migraine risk, of which *MEF2D*,* OSBPL10*,* B9D2*,* HTRA1* and *PRDM16* were classified as high- or medium-evidence genes. Notably, *PNKP* and *B9D2* demonstrated consistent cross‑tissue evidence across multiple molecular layers, including TWAS, gene expression, DNA methylation, and alternative splicing. *PNKP*, prioritized through integrative analyses of EDC-related genes and migraine-associated omics datasets, exhibited limited individual differential gene expression but significant enrichment of pathways related to hypothalamic–pituitary axis dysfunction, blood circulation, and pain signaling. Molecular docking and dynamics simulations further suggested stable binding between migraine-related target proteins and prioritized EDCs, including triphenyl phosphate (TPP), tris(1,3-dichloro-2-propyl) phosphate (TDCPP), and benzo(a)pyrene (B[a]P), providing preliminary connections between EDC exposure and migraine at the molecular level.

**Conclusions:**

This study provides integrative genetic and multi-omics evidence suggesting that EDC-related genes may be associated with to migraine susceptibility, highlighting the potential role of environmental factors to migraine risk. These findings identify a set of candidate EDCs and molecular targets that warrant further investigation, offering novel insights into migraine pathogenesis and informing future research on environmental determinants and preventive strategies for migraine.

**Graphical abstract:**

**Figure:**
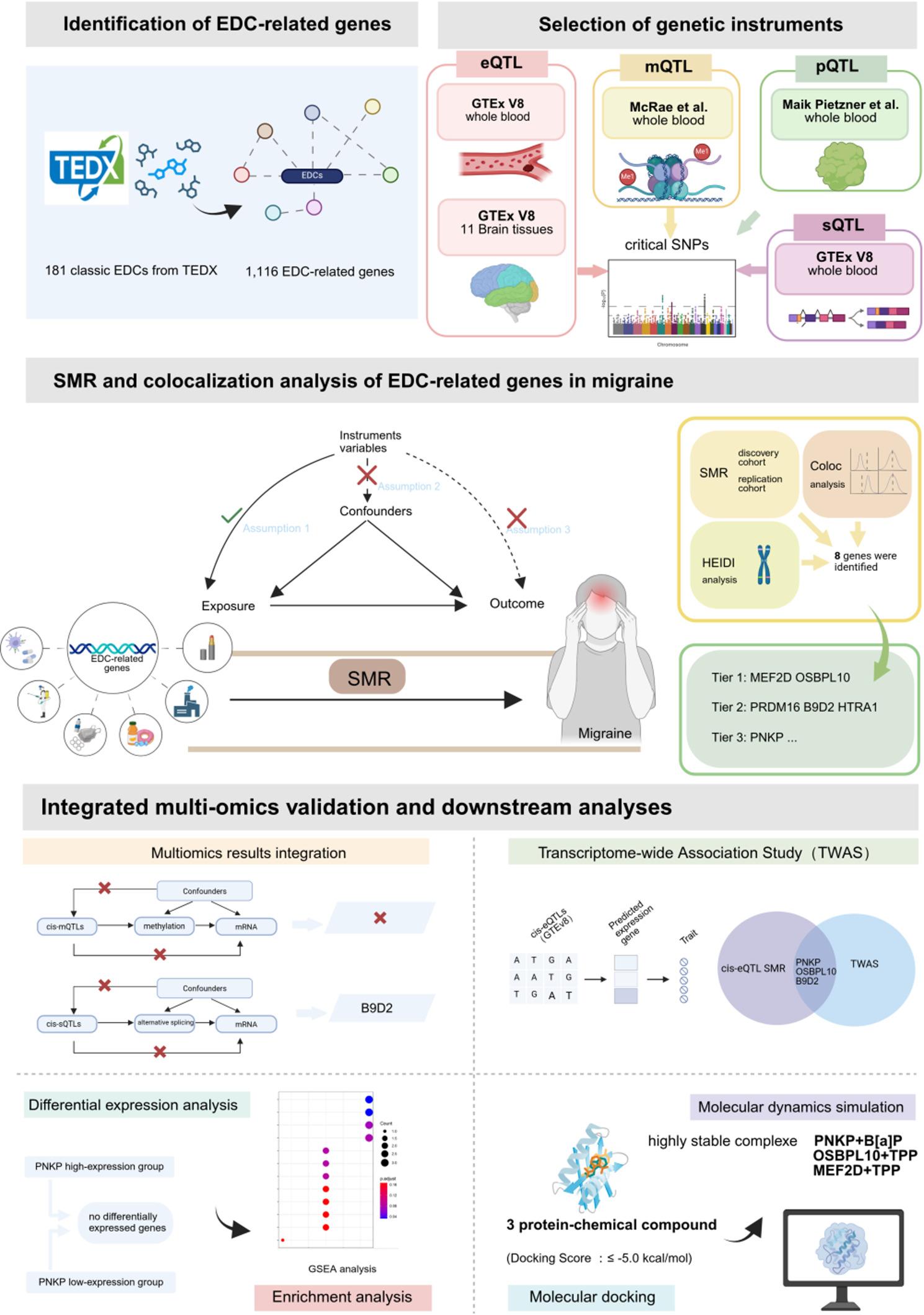

**Supplementary Information:**

The online version contains supplementary material available at 10.1186/s10194-026-02412-0.

## Background

Migraine is a common and disabling neurological disorder characterized by recurrent attacks of moderate to severe headache accompanied by neurological and systemic symptoms [1]. It ranks among the leading causes of years lived with disability worldwide [2], affecting more than one billion individuals, with an estimated annual prevalence of approximately 15% [3]. A marked female predominance is observed [4], with a female-to-male ratio of approximately 3:1, and heritability estimates of up to 57% highlight a substantial contribution of genetic susceptibility [5]. However, genetic factors alone do not fully explain disease risk, underscoring the important role of environmental influences and gene-environment interactions in migraine pathophysiology.

Endocrine-disrupting chemicals (EDCs) have emerged as a growing environmental concern due to their ability to interfere with hormonal signaling pathways by mimicking or antagonizing endogenous hormones [6]. These compounds are widely present in industrial products, food packaging, personal care items, and pesticides, making exposure nearly ubiquitous. EDCs have been implicated in a range of adverse health outcomes, including reproductive dysfunction, metabolic disorders, and hormone-related cancers [7–9]. Increasing evidence also suggests that EDCs may affect the nervous system, with studies linking exposure to neurodevelopmental alterations, cognitive impairment, and behavioral changes [6]. Experimental studies further demonstrate that certain EDCs, such as bisphenol A (BPA) and octylphenol (OP), can disrupt calcium signaling and cellular differentiation, providing biological plausibility for their role in neurological disorders [10].

Migraine exhibits a strong hormonal component, as reflected by its sex-specific prevalence and sensitivity to fluctuations in sex hormones. Estrogen withdrawal is a key factor in triggering menstrual migraine, whereas a relatively stable hormonal environment, such as during pregnancy, exerts a protective effect [11, 12]. Other neuroendocrine factors, including progesterone, androgens, oxytocin and pituitary adenylate cyclase-activating polypeptide (PACAP), modulate migraine susceptibility by influencing nociceptive signaling, trigeminovascular activation, and neuroinflammatory responses [11–14]. Given that many EDCs possess estrogenic or anti-estrogenic activity, it is biologically plausible that environmental exposure to these chemicals may influence migraine susceptibility, potentially through interactions with genetically regulated molecular pathways.

However, direct evidence linking EDCs to migraine remains limited. Traditional epidemiological studies are constrained by confounding, small sample sizes, and the difficulty in isolating the effects of individual chemicals from complex environmental mixtures. Nonetheless, few studies have systematically integrated genetic data to assess how EDC-related molecular mechanism contribute to migraine risk.

In this study, we applied an integrative analytical framework combining chemical-gene interaction data, large-scale genomic resources and causal inference approaches. Summary data-based Mendelian randomization (SMR), HEIDI testing, Bayesian colocalization, and transcriptome-wide association studies (TWAS) was employed to evaluate genetically predicted molecular traits of EDC-related genes in relation to migraine risk. Multi-omics integration characterized regulatory mechanisms across molecular layers, with molecular docking and dynamics simulations assessed EDC-protein interaction related to migraine. By integrating environmental, genetic, and multi-omics data, this study identifies molecular pathways involving genes previously reported to interact with EDCs that may be associated with migraine, suggesting possible targets for further investigation [15].

## Methods

### Study design

**Fig. 1:**
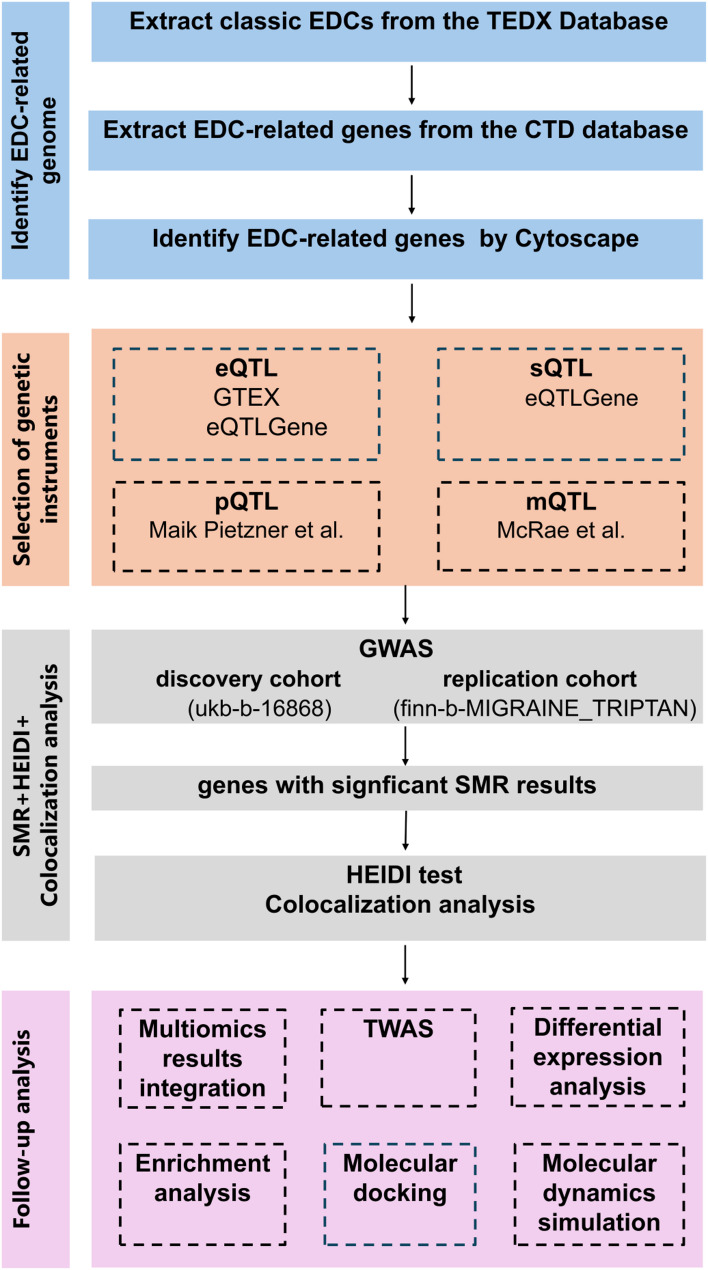
Study design and analytical workflow

This study employed a multi-step integrative approach to identify and validate EDC-related genes associated with migraine (Fig. 1). Candidate endocrine-disrupting chemicals (EDCs) were obtained from The Endocrine Disruption Exchange (TEDX) database (https://endocrinedisruption.org). Chemicals listed across 12 predefined categories and added between 2015 and 2018 were collected, yielding 389 candidate compounds. Human chemical-gene interaction data were subsequently retrieved from the Comparative Toxicogenomics Database (CTDB, https://ctdbase.org). To ensure the specificity of interactions and mitigate non-specific associations, interactions between EDCs and genes were restricted to *Homo sapiens* and to curated interactions involving gene expression, methylation, alternative splicing, or protein activity modulation. After filtering, 181 EDCs corresponding to 7,264 human genes were identified (Table S1). A chemical-gene interaction (CGI) network (Table S2) was constructed using Cytoscape (v3.10.2). Genes with degree centrality values ≥ 2× the network median were selected for downstream analyses [16], resulting in 1,116 candidate EDC-interacting genes (Table S3), as it was used to prioritize highly connected genes within the CGI network while reducing sparse and potentially non-specific interactions.

### Data sources of GWAS and QTL

Summary data for expression quantitative trait loci (eQTL) were obtained from blood samples in the eQTLGen Consortium (*n* = 31,684 individuals) [17] and GTEx V8 database, which includes genetic data for both blood and 12 types of brain tissue (*n* = 838 individuals) [18]. Splicing QTL (sQTL) blood data were obtained from the eQTLGen Consortium. Methylation QTL (mQTL) blood data were derived from a meta-analysis of two European cohorts: the Brisbane Systems Genetics Study (*n* = 614 individuals) and the Lothian Birth Cohorts (*n* = 1,366 individuals) [19]. Protein QTL (pQTL) blood data were obtained from a large-scale proteomic study by Pietzner et al. (*n* = 10,708 individuals) [20]. Based on harmonized summary statistics and ancestry compatibility with the European QTL resources used for integrative multi-omics analyses, the discovery GWAS data were obtained from the UK Biobank (OpenGWAS: ukb-b-16868), comprising 13,597 cases and 449,336 healthy controls. The replication GWAS data were derived from the FinnGen R10 (OpenGWAS: finn-b-MIGRAINE_ TRIPTAN), including 19,676 cases and 199,116 healthy controls [21].

### SMR and replication analysis

SMR uses genetic variants (e.g., SNPs) as instrumental variables, leveraging their random assortment property, to analyze the causal relationship between gene expression levels and disease. The validity of causal inference in SMR relies on three core assumptions: the relevance assumption, the independence assumption, and the exclusion restriction assumption. Previously identified EDC-related genes were intersected with cis-QTL data and analyzed using SMR software (version 1.3.1) [22]. The reference panel for linkage disequilibrium estimation was derived from European population data in the Complex Trait Genetics Lab database [23].

Cis-QTL instruments were selected within ± 1 Mb of each gene using a genome-wide significance threshold (*P* < 5 × 10^− 8^). SNPs with allele frequency differences > 0.2 between datasets (including the reference sample, cis-QTL summary data, and outcome summary data) were excluded, and the proportion of such SNPs was limited to < 5% for each QTL dataset. Multiple testing correction was performed separately for each QTL type using the Benjamini-Hochberg false discovery rate (FDR) method. Associations with FDR-corrected *P*_SMR_ < 0.05 were considered significant in the discovery stage and subsequently advanced to independent replication evaluation, heterogeneity in dependent instruments (HEIDI) tests and colocalization analyses. Independent migraine GWAS replication cohorts were applied to evaluate the significant association identified in the discovery stage using identical analytical parameters. Bonferroni correction was applied for replicated tests (*P*_SMR_ < 0.05/*n*), where *n* represents the number of significant associations identified from the discovery stage.

### HEIDI and colocalization analysis

Heterogeneity in dependent instruments (HEIDI) tests were performed to distinguish shared genetic associations from linkage disequilibrium effects. Associations with *P*_HEIDI_ >0.05 were retained for downstream analyses to reduce the risk of excluding potentially informative signals and further evaluated with colocalization analysis and evidence stratification. Bayesian colocalization analysis was performed using the *coloc* R package (version 5.2.3) to evaluate whether molecular traits of EDC-related genes and migraine share causal variants [24]. Colocalization analyses were conducted within a cis‑window of ± 1000 kb centered on each target gene for eQTL, sQTL, and pQTL datasets. For mQTL, analyses were restricted to a cis‑window of ± 500 kb centered on each CpG site. Five hypotheses (H0-H4) were tested [25]: H0, no association; H1, association with expression only; H2, association with migraine only; H3, independent associations; and H4, shared causal variants. Prior probability was set to 1 × 10^− 4^ for trait-specific associations and 1 × 10^− 5^ for shared causal variants. Regional association results were visualized using the *LocusCompare* R package (version 1.0.0) [26]. PPH4 ≥ 0.7 together with PPH4/(PPH3 + PPH4) ≥ 0.8 was used as the threshold for a shared causal variant [27].

Based on the combined evidence from SMR, HEIDI analyses and colocalization, identified associations were stratified into three confidence tiers, as previously reported [28]. Tier 1 loci passed all validation analyses, including SMR, HEIDI test, and colocalization analyses, with strong colocalization support (PPH4 > 0.7). Tier 2 loci passed most validation analyses but demonstrated medium colocalization support (PPH4 > 0.5) or *P*_HEIDI_ > 0.01. Tier 3 loci passed partial validation analyses, including SMR and HEIDI tests, but did not meet the predefined colocalization criteria.

### Multiomics results integration

To investigate regulatory relationships across molecular layers (methylation sites, alternative splicing, and gene expression), SMR analyses were performed using mQTL and sQTL as exposures and eQTL as outcomes. Analytical parameters and procedures were consistent with those used in the primary cis-QTL-GWAS analyses. Statistical significance was defined as FDR-corrected *P*_SMR_ < 0.05 and *P*_HEIDI_ > 0.05. Multiple testing correction was applied separately for each QTL type.

### Transcriptome-wide association study (TWAS) analysis

TWAS analysis was performed with the FUSION tool (http://gusevlab.org/projects/fusion/), integrating migraine GWAS data with eQTL data from GTEx V8 across six tissues, including whole blood, nucleus accumbens (basal ganglia), cerebellum, cortex, hippocampus, anterior cingulate cortex (BA24), to estimate gene-trait associations [29]. Linkage disequilibrium (LD) between SNPs was estimated using 1,000 Genomes European samples. FUSION integrates several predictive models (BLUP, BSLMM, LASSO, Elastic Net, and top 1) to estimate gene expression weights, with out-of-sample R² as the measure of predictive performance. The model achieving the highest cross-validation R² is selected as the final gene–tissue weight model, provided that its predictive performance is nominally non-zero (*P* < 0.05). Moreover, only genes with nominally significant (*P* < 0.05) estimates of cis-SNP heritability are retained [29, 30]. These gene weights were then integrated with GWAS summary statistics (Z-scores) to calculate TWAS association statistics. Statistical significance was defined as FDR-corrected *P* < 0.05. FDR was applied separately for each QTL type.

### Differentially expressed genes (DEGs) analysis

Raw gene expression data for migraine were obtained from the public database (E-MTAB-13397). This total RNA-seq dataset was generated from peripheral blood of *Homo sapiens* using the Illumina sequencing platform, and it comprises 44 migraine samples. Given that PNKP showed significant associations with migraine across multiple tissues, patients were stratified into *PNKP* high- and low-expression based on median expression level of *PNKP.* Differential expression analysis was performed in R (v4.2.1) using the limma-voom workflow following trimmed mean of M-values (TMM) normalization in edgeR, with DESeq2 additionally applied as a complementary count-based method. Significant DEGs were identified with thresholds of FDR < 0.05 and |log_2_ FC| > 1. Genes with log_2_ FC > 1 were defined as upregulated (Up), while those with log_2_ FC < -1 were categorized as downregulated (Down). All remaining genes were regarded as non-significant (NotSig).

To investigate pathway-level biological differences associated with PNKP expression, Gene Set Enrichment Analysis (GSEA) was performed using clusterProfiler (v4.10.0) based on ranked moderated t-statistics. Functional gene sets were obtained from the Molecular Signatures Database (MSigDB, v2023.2.Hs), including hallmark, WikiPathways, Reactome, KEGG hsa, and HPO collections. GSEA was conducted with 1,000 permutations and weighted enrichment statistics, restricting gene set size to 25–500 genes. Gene sets with |NES| ≥ 1.5 and FDR q-value < 0.05 were considered significantly enriched.

### Molecular docking

Molecular docking was performed using AutoDock Vina (v1.2.7) to predict the binding between EDCs and target proteins. Ligand structures of EDCs were obtained from the PubChem database and converted with Open Babel (v3.1.1). Protein structures were retrieved from the RCSB PDB database (http://www.rcsb.org/), with high-resolution crystal structures prioritized [31, 32].

Protein preparation was performed in PyMOL (v3.1) by removing water molecules and non-essential ligands, followed by hydrogen addition. Ligand structures were energy-minimized using ChemDraw (v20.0), and PDBQT files were generated with AutoDock Tools (v1.5.6). Docking grid box was set to cover the entire protein structure. Among nine generated conformations, the pose with the lowest binding energy and highest clustering frequency was selected as the most probable binding mode and visualized in PyMOL.

### Molecular dynamics simulation

Molecular dynamics (MD) simulations were performed using GROMACS 2022.2 [33]. Protein parameters were generated using the CHARMM36 force field [34]. For ligand small molecules, topology files, bonded parameters, and non‑bonded parameters compatible with the CHARMM36 force field were obtained via the CGenFF server to ensure force field consistency between the ligand and the receptor [35]. Atomic partial charges of the ligand were assigned according to the standard CHARMM fitting protocol. Systems were solvated using TIP3P water molecules with a minimum protein–box distance of 1.0 nm. Na⁺ and Cl⁻ ions were added to neutralize the system and maintain a physiological ionic strength of 0.15 M NaCl. Long-range electrostatic interactions were treated using the Particle Mesh Ewald (PME) method with a cutoff of 1.0 nm, and bond constraints were handled using the LINCS algorithm. Following 3,000 steps of steepest descent and 2,000 steps of conjugate gradient energy minimization, systems underwent NPT equilibration and production simulations with a 2 fs integration time step. Temperature was maintained at 310 K using a Nosé-Hoover thermostat, and pressure was maintained at 1 bar using a Parrinello-Rahman barostat. Position restraints were applied during equilibration and subsequently removed prior to production runs. To improve conformational sampling robustness and reproducibility, three independent 100 ns replicate simulations were performed for each system.

Trajectory analyses were conducted using GROMACS utilities to calculate root-mean-square deviation (RMSD), root-mean-square fluctuation (RMSF), hydrogen bonds, radius of gyration (Rg), solvent-accessible surface area (SASA), and free energy landscape (FEL). Principal component analysis (PCA) based on the first two principal components was integrated with FEL analysis to characterize conformational distributions, energetic preferences, and overall system stability.

## Results

### SMR analysis of EDC related-genes in discovery cohort

**Fig. 2:**
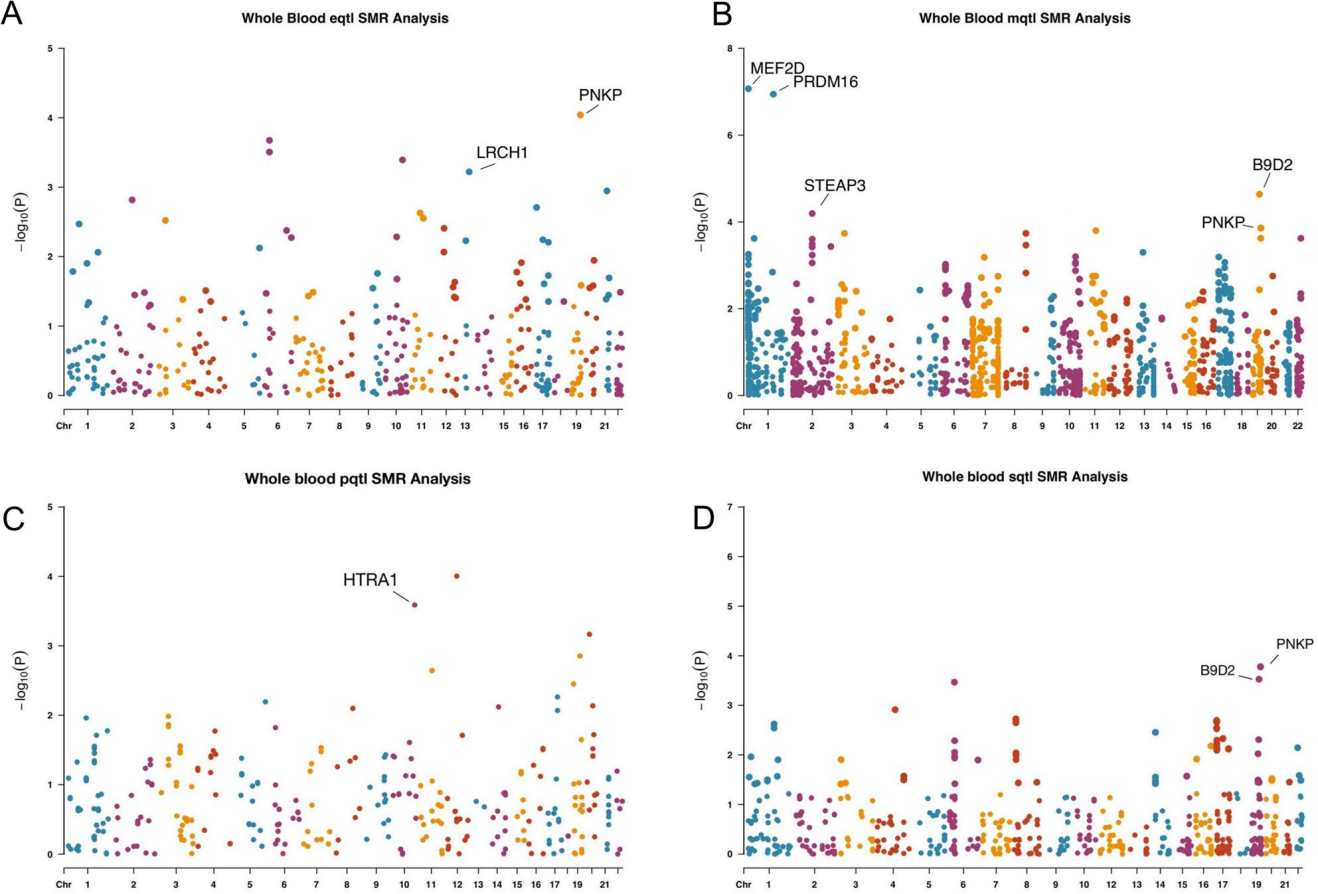
Manhattan plot illustrating genes associated with migraine. Manhattan plot of SMR associations for migraine based on whole blood **(A)** eQTL, **(B)** mQTL, **(C)** pQTL, **(D)** sQTL datasets. The x‑axis shows the chromosomal position of the tested genes, arranged from chromosome 1 to 22, with alternating colors used to distinguish adjacent chromosomes. The y‑axis represent the SMR *P*‑value (-log_10_). Labeled genes indicate prioritized significant associations

**Fig. 3:**
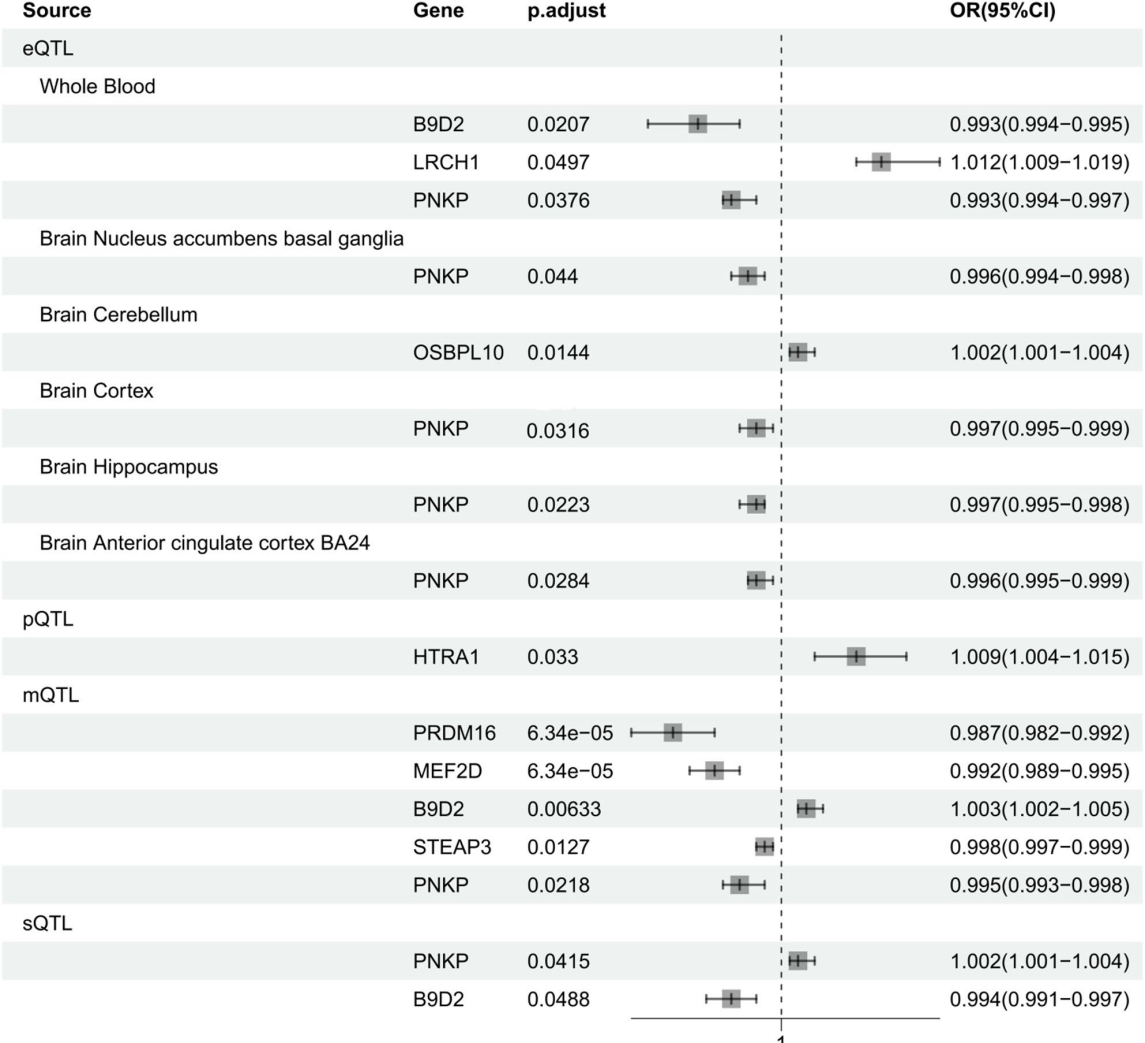
SMR results for the association between the EDCs-related genes and migraine in discovery cohort. Forest plot showing effect estimates (odds ratios, ORs) and 95% confidence intervals for genes identified by SMR analysis across eQTL, pQTL, mQTL, and sQTL datasets. Results are presented for whole blood and brain tissues where applicable. p.adjust, FDR-adjusted P value from the SMR test; CI, confidence interval; OR, odds ratio

4 EDC-related genes across 6 tissues (blood, cerebellum, nucleus accumbens, cortex, hippocampus, and anterior cingulate cortex BA24) were significantly associated with migraine in cis-eQTL-based SMR analysis (FDR < 0.05; Fig. 2A; Table S4-10). For instance, *PNKP* (OR = 0.994; 95% CI: 0.992–0.997) and *B9D2* (OR = 0.990; 95% CI: 0.984–0.995) were inversely associated with migraine, whereas *LRCH1* (OR = 1.012; 95% CI: 1.005–1.019) and *OSBPL10* (OR = 1.009; 95% CI: 1.004–1.015) were positively correlated with migraine risk. Notably, *PNKP* showed consistent associations across blood and multiple brain regions (Fig. 3B).

In mQTL analysis, 5 EDC-related genes were associated with migraine (Fig. 2B; Table S11). Methylation at *PRDM16* (OR = 0.987; 95% CI: 0.982–0.992), *MEF2D* (OR = 0.992; 95% CI: 0.989–0.995), *STEAP3* (OR = 0.998; 95% CI: 0.997–0.999), and *PNKP* (OR = 0.995; 95% CI: 0.993–0.998) was inversely associated with migraine risk, whereas methylation at *B9D2* (OR = 1.003; 95% CI: 1.002–1.005) showed positive associations.

**Fig. 4:**
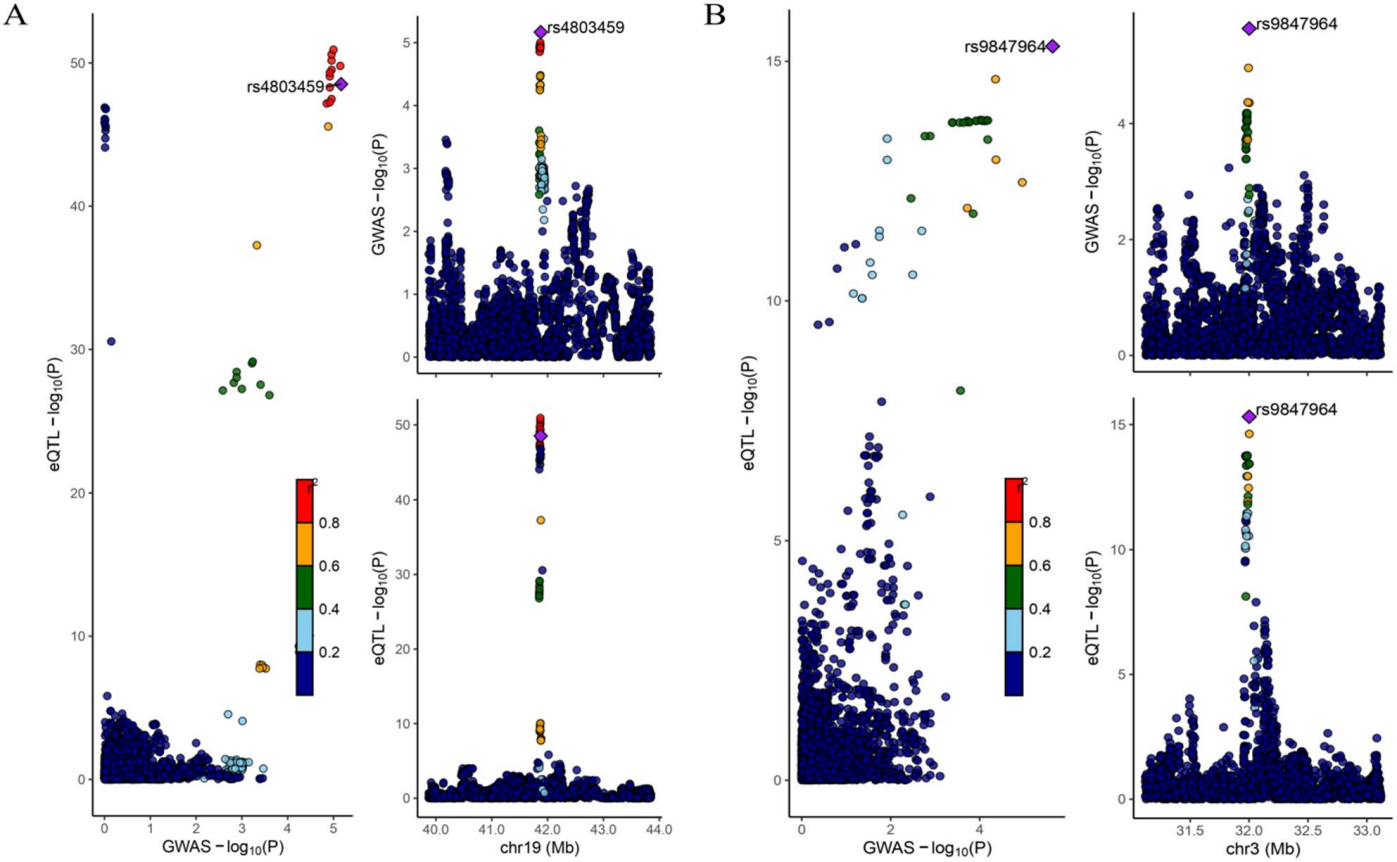
Regional colocalization plots for EDC-related genes and migraine. Colocalization of B9D2 (**A**) and OSBPL10 (**B**) with migraine. Each panel displays regional association signals for GWAS and eQTL data, with SNPs colored by linkage disequilibrium relative to the lead variant

In pQTL analysis, only one protein was associated with migraine (Fig. 2C; Table S12). The abundance of *HTRA1*(OR = 1.009; 95% CI: 1.004–1.015) showed negative correlations (OR < 1). sQTL analysis showed that alternative splicing of *PNKP* (OR = 1.002; 95% CI: 1.001–1.004) was positively associated with migraine, whereas alternative splicing of *B9D2* (OR = 0.994; 95% CI: 0.991–0.997) showed an inverse association (Fig. 2D; Table S13). All results of the eQTL-GWAS, mQTL-GWAS, pQTL-GWAS, and sQTL-GWAS analyses are summarized in figure (Fig. 4).

In the replication cohort, the 2 out of 16 significant associations identified in the discovery stage were subjected to validation. A stringent Bonferroni correction threshold of *P* < 0.05/16 (≈ 3.13 × 10⁻³) was applied. A total of 2 associations in cis‑mQTL analysis were successfully replicated, with effect directions consistent with the discovery analysis. Methylation levels at *PRDM16* (*P* = 7.76 × 10^− 6^; OR = 0.729; 95% CI: 0.635–0.837) and *MEF2D* (*P* = 8.89 × 10^− 4^;OR = 0.872; 95% CI: 0.837–0.945) were both inversely associated with migraine risk (Table S14).

### HEIDI test and Colocalization analysis

**Fig. 5:**
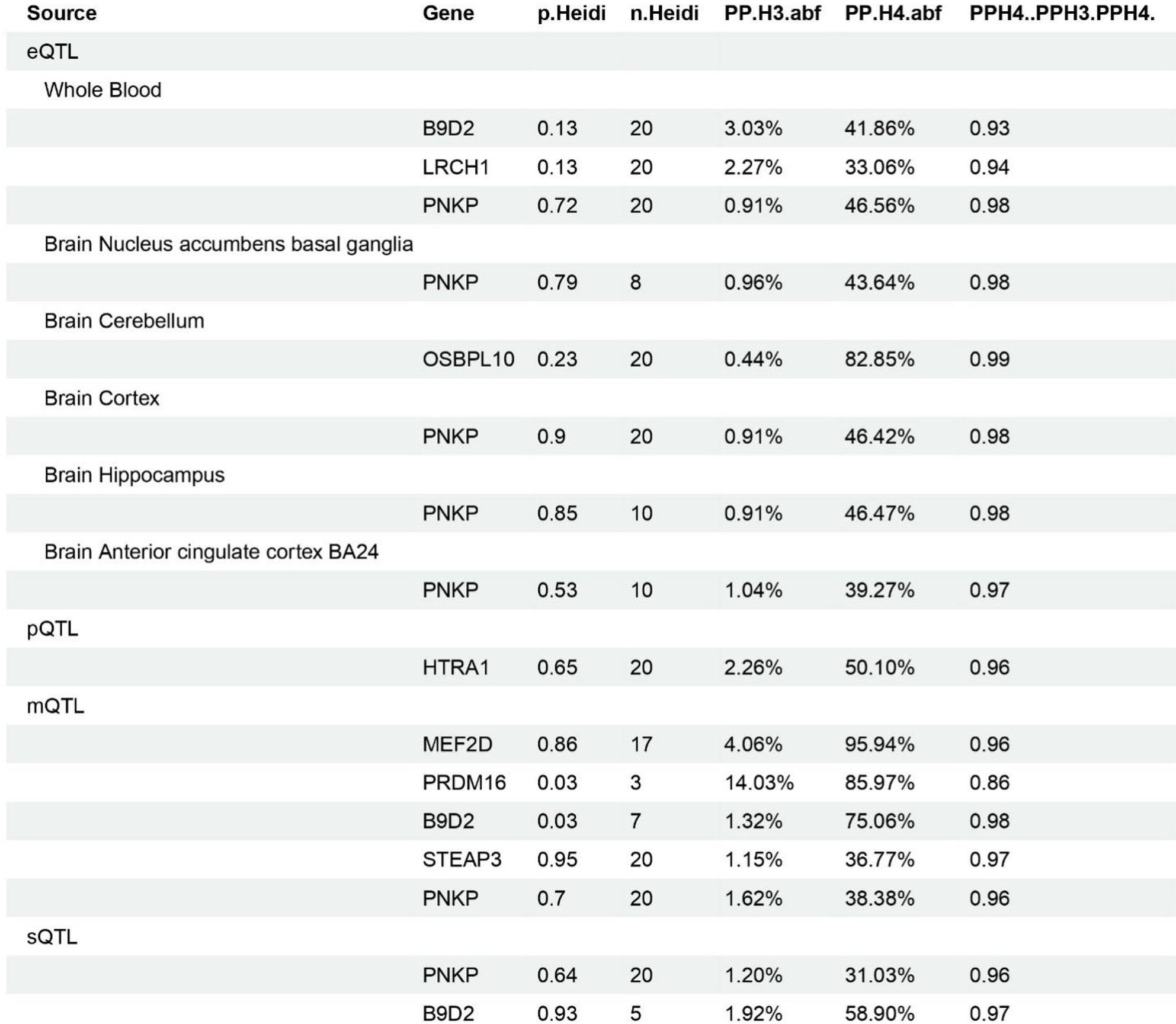
Results of HEIDI test and colocalization analysis. p.Heidi P value from the HEIDI test; n.Heidi, number of SNPs used in the HEIDI test; PP.H3, posterior probability for hypothesis 3 (independent signals); PP.H4, posterior probability for hypothesis 4 (shared causal variant)

Among the significant associations, most loci passed the HEDI test (*P*_HEIDI_), except for *PRDM16* (*P*_HEIDI_=0.03) and *B9D2* (*P*_HEIDI_=0.03), which were slightly below the threshold. In colocalization analysis, *OSBPL10* (PPH4 = 0.8285, PPH4/(PPH3 + PPH4) = 0.99; Fig. 5B) in cerebellar eQTL, *MEF2D* (PPH4 = 0.9594, ratio = 0.96), PRDM16 (PPH4 = 0.8597, ratio = 0.86) and *B9D2* (PPH4 = 0.7506, ratio = 0.98; Fig. 5A) in mQTL showed the strongest support for colocalization. *HTRA1* (*P*_HEIDI_=0.65, PPH4 = 0.5010, ratio = 0.96) in pQTL showed moderate colocalization support. Other loci did not pass the test of colocalization analysis.

**Fig. 6:**
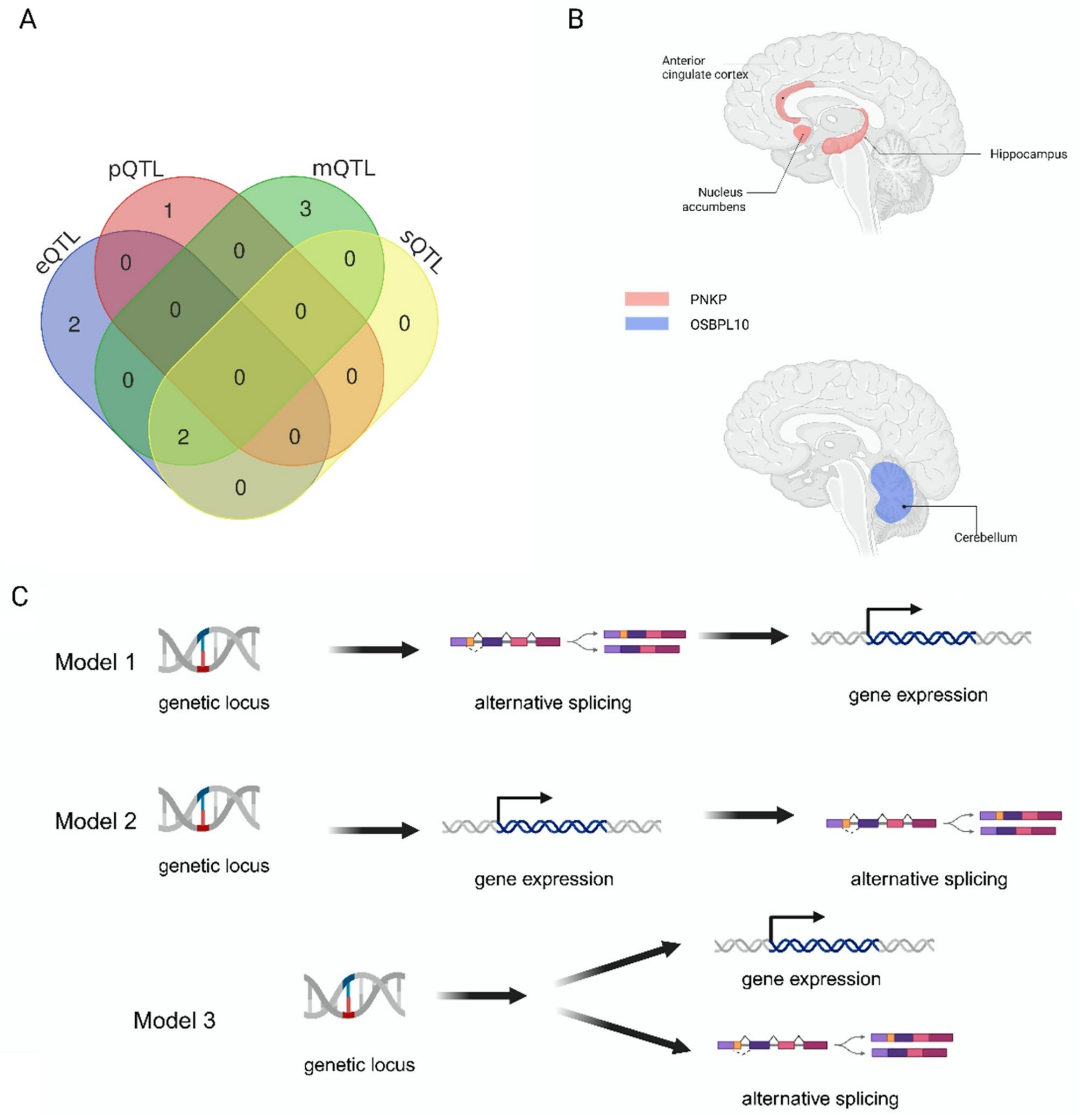
Multi-omics integration of EDC-related genes associated with migraine. (**A**) Venn diagram showing overlap of genes with significant SMR associations across eQTL, mQTL, pQTL, and sQTL datasets. (**B**) Tissue distribution of representative genes (PNKP and OSBPL10) with significant SMR associations across brain regions. (**C**) Model of the association between B9D2 alternative splicing and gene expression mediated by a shared genetic locus. Graphics was created with BioRender.com

By combining the results from SMR, HEIDI test and colocalization analysis, we classified the identified gene loci based on evidence strength. *MEF2D*, *OSBPL10* passed all analyses and was classified as Tier (1) *PRDM16* and *B9D2* which were slightly below the threshold of HEIDI test, and *HTRA1* which showed moderate colocalization support were classified as Tier (2) Other loci that did not pass the HEIDI test or colocalization analysis were classified as Tier (3) The results of HEIDI test and colocalization analysis are summarized in figure (Fig. 6).

### Multi-omic integration reveals genes regulatory mechanisms in migraine

Multi-omics integration identified *PNKP* and *B9D2* as genes associated with migraine across methylation, alternative splicing, and gene expression layers (Fig. 3A). sQTL-eQTL analysis showed that alternative splicing of *B9D2* was positively associated with its gene expression (OR = 1.219; 95% CI: 1.092–1.361; Table S15). This indicates the presence of a shared genetic variant between them, that is, a genetic locus that is associated with both *B9D2* splicing and total expression. This locus may regulate B9D2 function by affecting splicing, expression, or a combination of both. However, the causal direction of this regulation cannot be inferred. In reality, reverse regulation or co-regulation by upstream factors may exist (Fig. 3C). mQTL-eQTL analysis did not support an association between methylation and gene expression for *PNKP* or *B9D2* (Table S16).

**Fig. 7:**
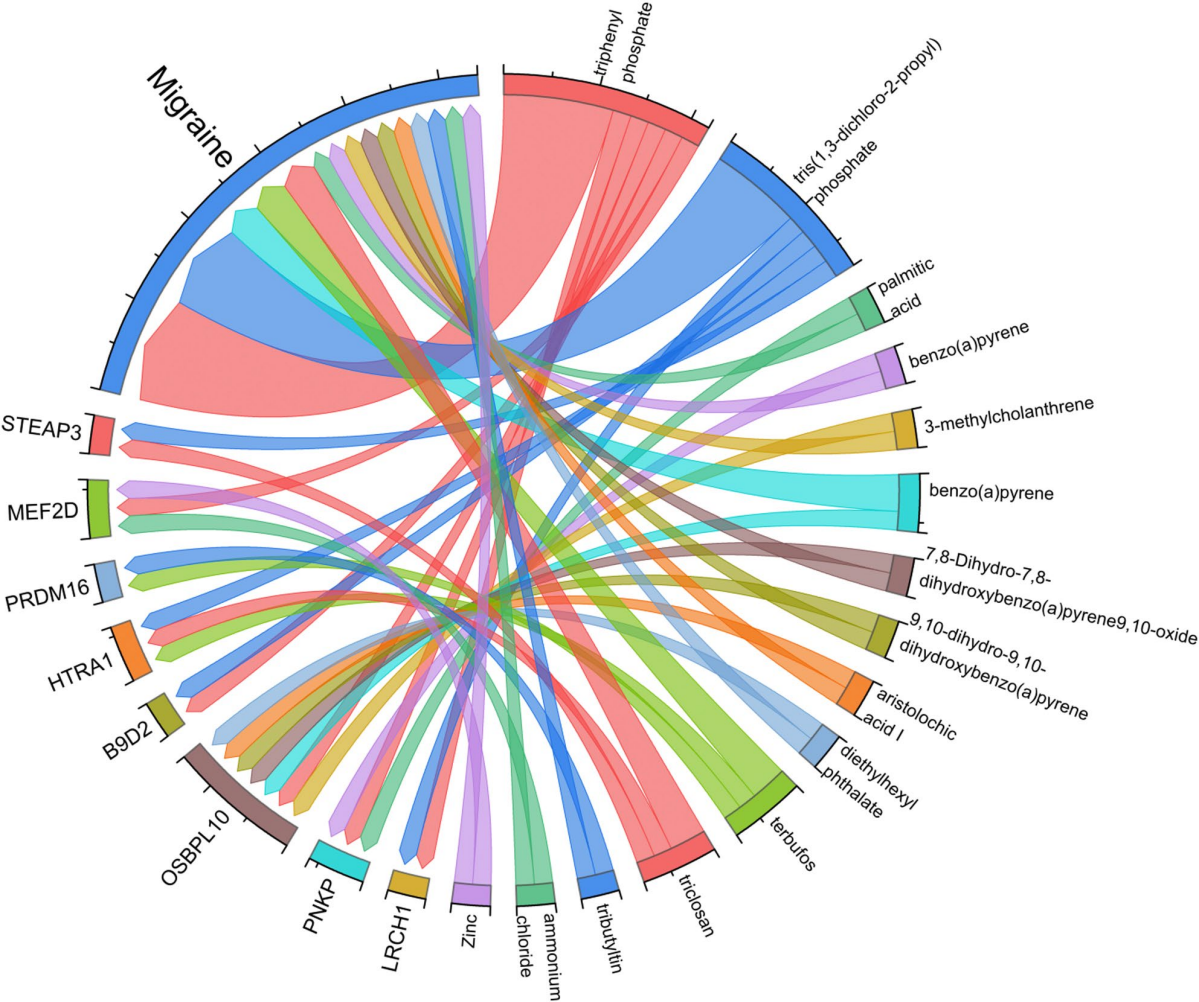
Chord diagram of interactions among EDCs, key genes, and migraine. Chord diagram illustrating relationships among EDCs, prioritized genes identified by SMR analysis, and migraine. Colored ribbons represent curated chemical–gene interactions retrieved from the Comparative Toxicogenomics Database (CTD), linking individual EDCs to associated genes. Gene-migraine associations were inferred from SMR analyses. Ribbon width is proportional to the number of reported interactions

To characterize environmental links, the 16 prioritized associations (corresponding to 8 genes) were mapped to corresponding EDCs using curated data from CTD and TEDX (Table S23). The resulting chord diagram illustrates interactions among EDCs, genes, and migraine (Fig. 7). Several EDCs, including triphenyl phosphate and tris(1,3-dichloro-2-propyl) phosphate were linked to multiple genes, among which *OSBPL10* and *PNKP* showed interactions with multiple EDCs, indicating their potential as key molecular hubs within the EDC-gene-migraine network.

### TWAS validates EDC-related genes across tissues and brain regions in migraine

**Fig. 8:**
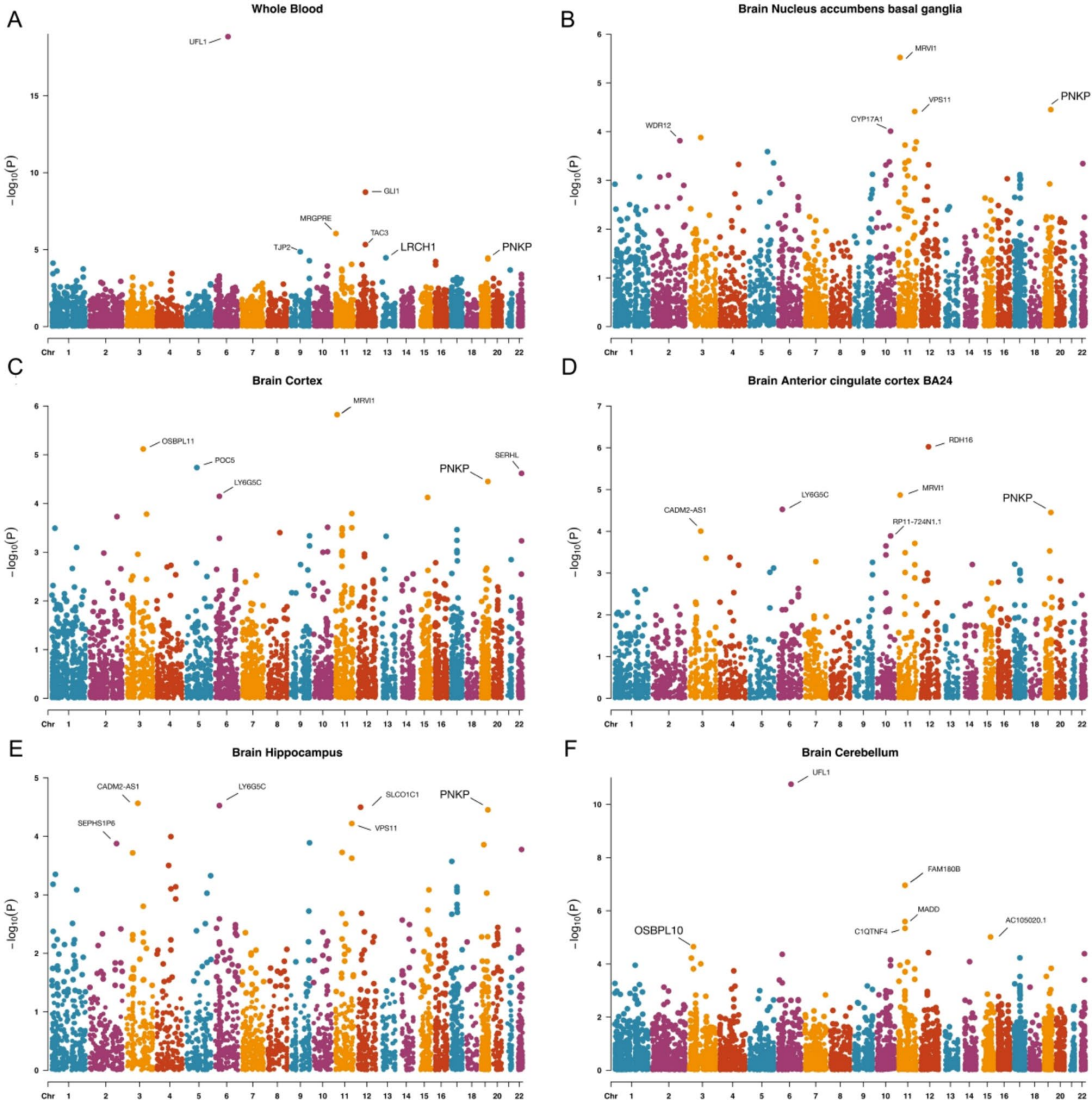
Manhattan plots of TWAS-validated genes associated with migraine across tissues. (**A**) Whole blood. (**B**) Nucleus accumbens (basal ganglia). (**C**) Cortex. (**D**) Anterior cingulate cortex (BA24). (**E**) Hippocampus. (**F**) Cerebellum. Each point represents a gene, plotted by chromosomal position against -log₁₀(P) from TWAS analysis. Selected significant genes are labeled

SMR analysis identified EDC-related genes with shared genetic effects on gene expression and migraine risk, which were further validated using FUSION-based transcriptome-wide association analysis (TWAS) using genome-wide eQTL data from GTEx V8. In total, 33 genes reached significance (FDR < 0.05) in at least one tissue (Table S17-22). Among these, three genes overlapped with SMR results, including *PNKP*, *OSBPL10*, and *LRCH1* (Fig. 8). *PNKP* showed associations across multiple tissues, including whole blood, nucleus accumbens (basal ganglia), hippocampus, and anterior cingulate cortex (BA24). *OSBPL10* was associated with migraine in cerebellum and cortex, whereas *LRCH1* was identified in TWAS but showed more limited tissue distribution. These results provide cross-method consistency for a subset of EDC-related genes. *B9D2*, which was identified exclusively through eQTLGen in the SMR analysis, was not identified in the TWAS analysis.

### Transcriptomic and functional analyses reveal associated pathways

To characterize *PNKP*-related transcriptional changes, samples from the E-MTAB-13,397 dataset were stratified into high- and low-expression groups based on the median *PNKP* expression level. No statistically significant differentially expressed genes were identified between the two groups under conventional thresholds (|log2FC| > 1, adjusted *P* < 0.05), suggesting that variation in PNKP expression was not associated with large-scale transcriptomic perturbations in this cohort. The relatively limited sample size and homogeneous clinical characteristics of the E-MTAB-13,397 cohort may have further reduced the statistical power to detect subtle expression differences.

**Fig. 9:**
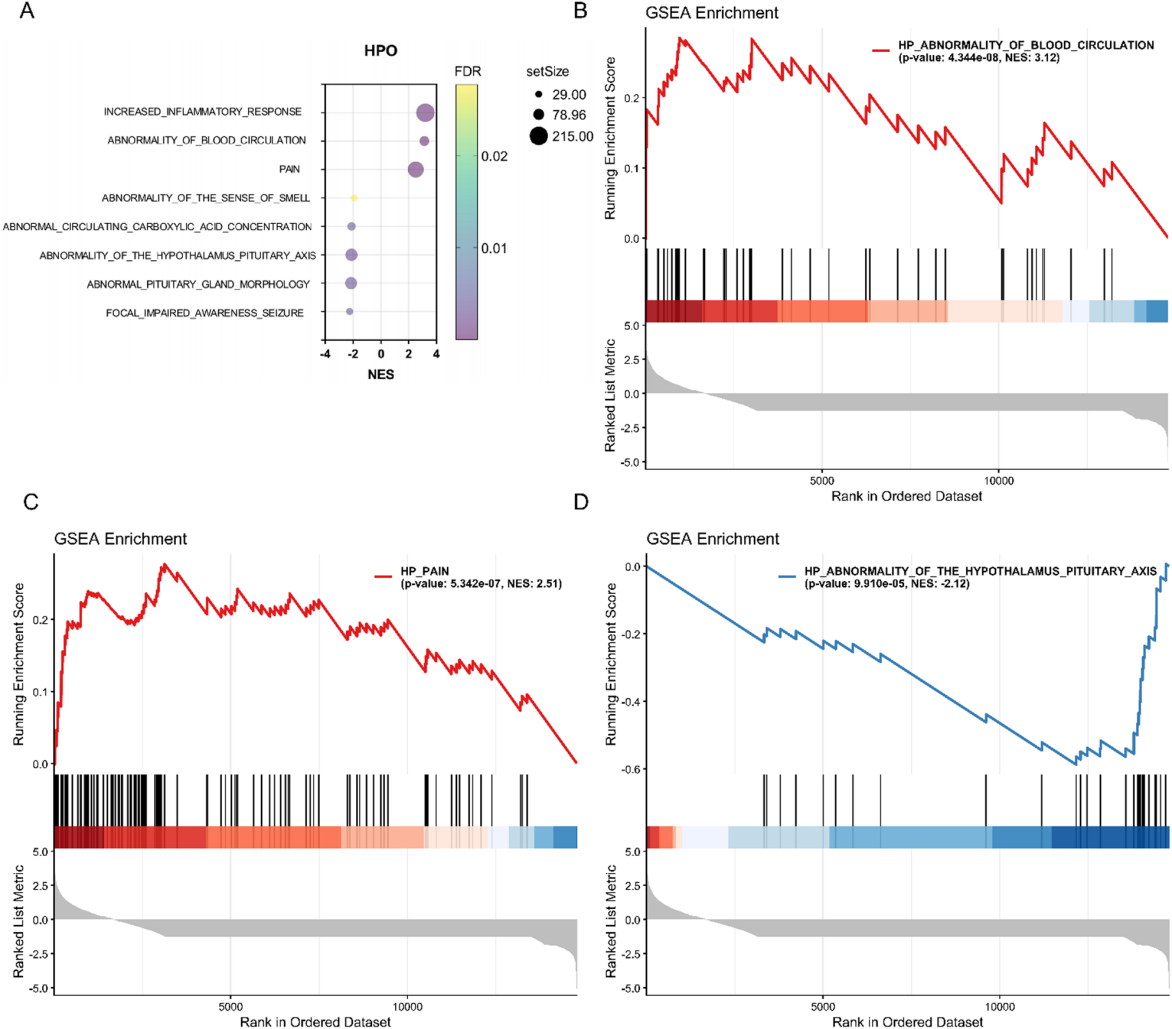
GSEA enrichment analysis of pathways associated with PNKP expression status. The bubble plot (**A**) shows the pathways associated with migraine across different PNKP expression groups, which were screened from the HPO database. “HP_ABNORMALITY_OF_ BLOOD_CIRCULATION” (NES = 3.12, p = 4.34×10-8) (**B**) and “HP_PAIN” (NES = 2.51, p = 5.34×10-7) (**C**) are positively enriched in the PNKP-high group, while “HP_ABNORMALITY_OF_THE_HYPOTHALAMUS_PITUITARY_AXIS” pathway (NES = -2.12, p = 9.91×10-5) are negatively enriched in the PNKP-high group (**D**).Vertical lines (barcode) mark gene positions; the histogram shows the ranking metric; gray shading indicates permutation-based score distributions. P, p-value; NES, normalized enrichment score

GSEA identified 515 significantly enriched gene sets. Many pathways have been studied and proven to be closely associated with migraine, for example, in the HPO database (Fig. 9A), the “ABNORMALITY _OF_THE_HYPOTHALAMUS_PITUITARY_AXIS” pathway was significantly downregulated in the PNKP-high group (NES = -2.11, FDR *q* = 0.003), with core enrichment genes including SIX3, SOHLH1, and SOX3 (Fig. 9D). In contrast, the “ABNORMALITY_OF_BLOOD_CIRCULATION” (NES = 3.12, FDR *q* = 7.77 × 10⁻⁶) and “PAIN” (NES = 2.51, FDR *q* = 5.27 × 10⁻⁵) pathways were significantly upregulated in the PNKP-high group (Fig. 9B-C), with core enrichment genes including *CFHR1*, *GATA6*, *F7* and *GATA6*,* PKDCC*,* TNFRSF11A.* These pathways have previously been implicated in migraine pathophysiology and may provide additional insight into the potential relationship between PNKP and migraine [36, 37].

### Molecular simulations support EDC-target interactions implicated in migraine

**Fig. 10:**
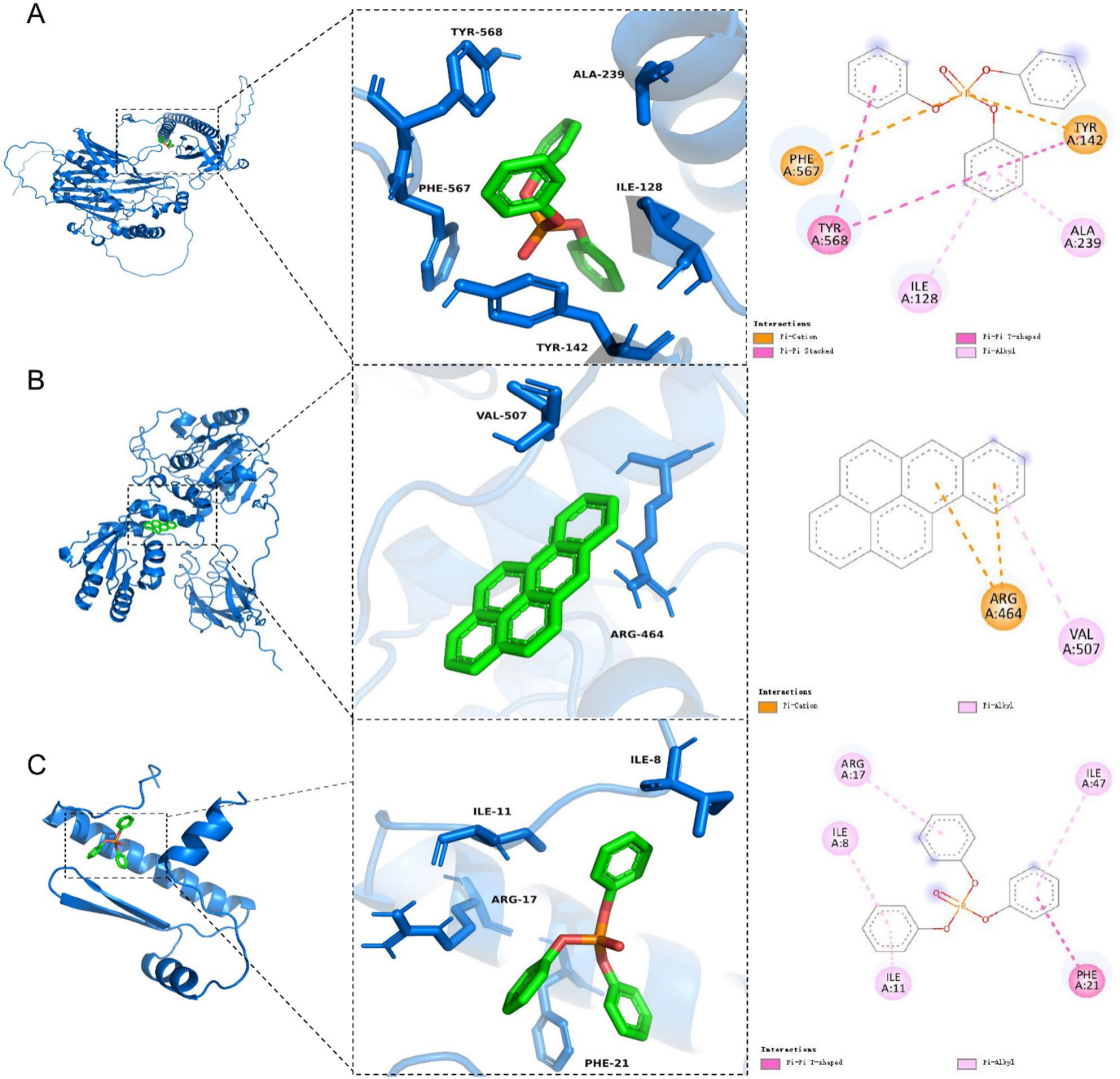
Molecular docking of EDCs with target proteins encoded by migraine-related genes. The left panel shows the overall docking conformation, the middle panel presents a close-up view of the binding site, and the right panel illustrates the 2D schematic of ligand-protein interactions for OSBPL10-TPP (**A**), PNKP- B[a]P (**B**), MEF2D-TPP (**C**). OSBPL10, oxysterol-binding protein-like protein 10; TPP, triphenyl phosphate; PNKP, Polynucleotide Kinase 3'-Phosphatase; B[a]P, benzo(a)pyrene; MEF2D, myocyte enhancer factor 2D

A total of 24 identified EDC-protein complexes were evaluated by molecular docking. Among them, triphenyl phosphate (TPP) and benzo[a]pyrene (B[a]P) demonstrated favorable binding affinities with several migraine-associated targets (binding energy < -5 kcal/mol) (Table S24), including triphenyl phosphate with myocyte enhancer factor 2D (TPP-MEF2D, -6.818 kcal/mol; Fig. 10C), triphenyl phosphate with oxysterol-binding protein-like protein 10 (TPP-OSBPL10, -6.53 kcal/mol; Fig. 10A), and benzo(a)pyrene with Polynucleotide Kinase 3’-Phosphatase (B[a]P-PNKP, -9.648 kcal/mol; Fig. 10B).

**Fig. 11:**
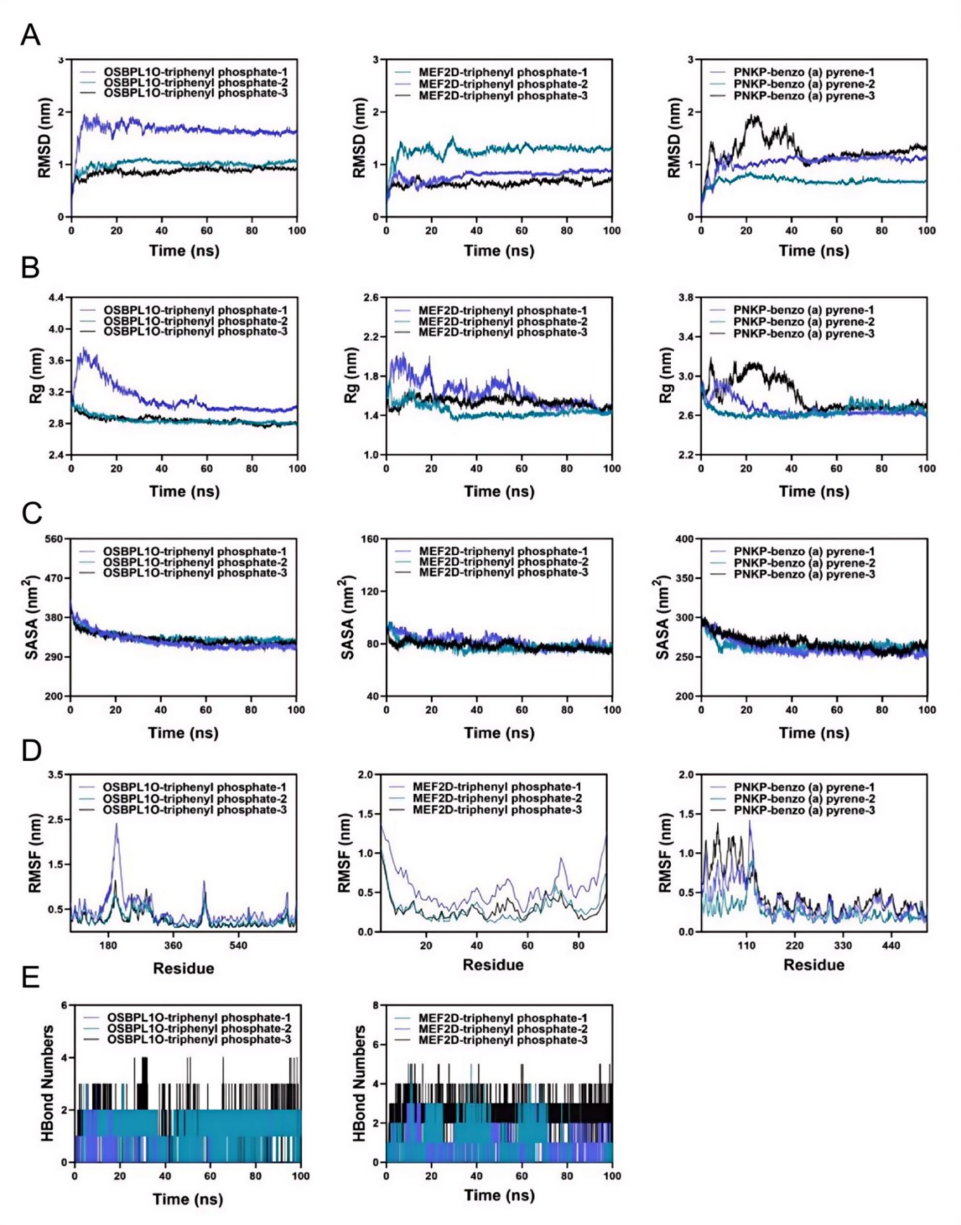
Molecular dynamics simulation of EDC-protein complexes related to migraine. Molecular dynamics simulations were performed for representative EDC-protein complexes, including PNKP-B[a]P, OSBPL10-TPP, and MEF2D-TPP, with three independent replicate simulations conducted for each complex. **A**. Root mean square deviation (RMSD). **B**. Radius of gyration (Rg). **C**. Solvent-accessible surface area (SASA). D. Root mean square fluctuation (RMSF). E. Number of hydrogen bonds over time

In three independent 100 ns molecular dynamics simulations, the OSBPL10-triphenyl phosphate, MEF2D-triphenyl phosphate, and PNKP-benzo(a)pyrene complexes all exhibited overall conformational stability but showed significant differences in dynamic behavior and equilibration characteristics (Fig. 11). The OSBPL10 complex displayed the strongest stability, with the smallest RMSD fluctuations (1.0–1.5 nm), reduced Rg and SASA indicating compact structure formation, relying mainly on hydrophobic interactions and exhibiting very low flexibility in core binding-pocket residues. The MEF2D complex showed moderate stability, with RMSD fluctuations of 1.0–1.8 nm, more persistent hydrogen-bond interactions, and overall stable conformation despite some N-terminal flexibility. The PNKP complex was the least stable, with RMSD initially rising to 2.0–2.5 nm before stabilizing at 1.5–2.0 nm, and higher flexibility in multiple loop and non-pocket regions, reflecting greater conformational dynamics. High reproducibility was observed across the three replicate runs.

**Fig. 12:**
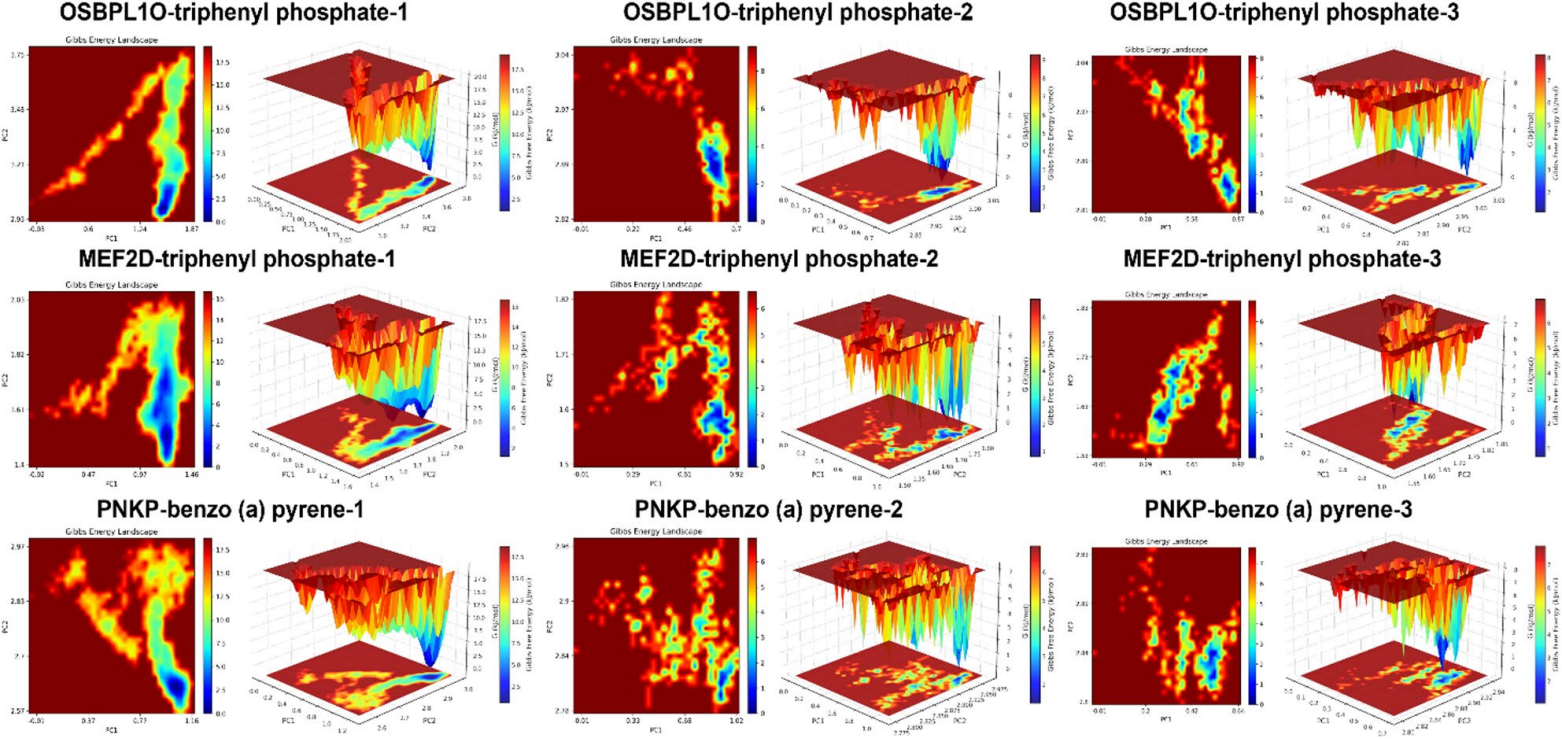
Free energy landscape diagram of EDC-protein complexes related to migraine. The figure shows the free energy landscapes of the three complexes. The x-axis represents the first principal component (PC1), capturing the dominant motion mode of the protein conformation. The y-axis represents the second principal component (PC2), describing secondary conformational changes. The colour scale indicates the free energy change, ranging from blue (lowest energy, most stable) through green, yellow to red (highest energy, unstable). For each pair, three independent replicate simulations were conducted, and the results were highly reproducible and consistent

Figure 12 shows the free energy landscapes of the three complexes (Fig. 12). The OSBPL1O complex exhibited a concentrated, single deep energy well across all replicates, indicating a dominant stable conformation. The MEF2D complex showed relatively dispersed energy basins, suggesting multiple conformational substates with similar energies and weaker stability than OSBPL1O. The PNKP complex displayed a scattered energy distribution with no clearly dominant conformation. Collectively, these findings provide preliminary structural evidence supporting stable interactions between prioritized EDCs and migraine-associated target proteins.

## Discussion

In this study, we systematically investigated the potential relationship between EDC-related genes and migraine through integrative genomic analysis. We identified 8 EDC-related genes significantly associated with migraine risk, of which 2 showed high-evidence, supporting shared genetic signals underlying gene expression, methylation, protein abundance and disease susceptibility. These findings were further validated by TWAS and supported by multi-omics integration, which revealed associations between DNA methylation, alternative splicing, gene expression, and migraine-related molecular traits [38].

Several previously reported genes provide biologically plausible links between EDC exposure and migraine. *HTRA1* influences migraine risk through two independent mechanisms: a common variant (rs2672592) reduces circulating HTRA1 levels, lowering risk, whereas rare protease-impairing mutations increase risk of migraine with aura, reflecting a dual “low-level protection and low-activity induction” mode [39]. STEAP3, a ferrireductase involved in iron homeostasis, modulates intracellular iron levels and TLR4-NF-κB inflammatory signals; its dysregulation promotes iron-dependent ROS accumulation and neuroinflammation, processes implicated in migraine susceptibility [40]. Our study provides support for the notion altered expression of genes previously reported to interact with EDCs may represent a key mechanism underlying their potential association with migraine. However, the association of some genes identified in our study, such as PRDM16, LRCH1, and B9D2, with migraine still requires independent validation.

Mapping gene-chemical interactions further highlighted several high-priority EDCs as potential environmental contributors to migraine. These compounds are known to disrupt endocrine signaling, induce oxidative stress, and affect vascular and neuronal function. TPP disrupts sex hormone synthesis, raising estradiol and testosterone levels, and together with its metabolites crosses the blood-brain barrier to cause neuronal damage and abnormal myelination [41–43]. TDCPP interferes with endocrine signaling and vascular development by inhibiting the Nrf2 and VEGF pathways, and induces neurodevelopmental toxicity via oxidative stress, neuroinflammation, mitochondrial damage, and epigenetic dysregulation [44, 45]. Other EDCs such as B[a] and BPA alter hormone-related receptor gene expression and cause developmental toxicity [46]. However, no study to date has been able to demonstrate that these EDCs can affect migraine through the above pathways.

Molecular docking and dynamics simulations provided additional mechanistic support by demonstrating stable interactions between prioritized EDCs and migraine-related target proteins. TPP and B[a]P exhibited stable binding to proteins encoded by migraine-associated genes, suggesting a potential key role in migraine pathogenesis. PNKP is a critical DNA repair enzyme for DNA single-strand breaks induced by oxidative stress. B[a]P is predicted to bind stably within its substrate-binding pocket, potentially interfering with its function. Its dysfunction may lead to persistent DNA damage, PARP-1 overactivity and NAD^+^ depletion, causing energy imbalance. These processes can activate TRPM2/NLRP3 neuroinflammation [47, 48], sensitizing the trigeminovascular system sensitization. OSBPL10 is a lipid-transfer protein involved in lipid metabolism and intracellular signaling [49–51]. TPP binding may impair its function, potentially disrupting lipid homeostasis and associated neuronal and inflammatory processes relevant to migraine. However, the results of molecular docking and molecular dynamics simulations have not been experimentally validated. These analyses are exploratory and hypothesis‑generating in nature, and the relationship between EDCs and migraine requires further investigation.

This study supports a role for EDCs in migraine through gene-environment interactions affecting hormonal signaling and downstream molecular pathways [52–54]. However, several limitations should be acknowledged. First, due to the limited availability of exposure-linked datasets, our analyses focused on genes previously reported to interact with EDCs rather than direct exposure-disease relationships, precluding causal inference. In addition, the degree centrality ≥ 2× median cutoff used in the CGI network was an empirical prioritization strategy that may have excluded biologically relevant low-connectivity genes and introduced network bias. Second, the discovery GWAS dataset had a relatively early release date and modest sample size, potentially reducing statistical power for low-frequency variants and weak-effect loci, while the limited overlap between SMR and TWAS findings may reflect differences in eQTL reference datasets and analytical frameworks. Third, the SMR framework and HEIDI test cannot fully distinguish causality, pleiotropy, and linkage disequilibrium, leaving potential residual bias. Moreover, the more lenient Tier 2 and Tier 3 criteria may have introduced false-positive associations and should therefore be interpreted cautiously as exploratory findings. Finally, the predominantly European ancestry of the included datasets may limit the generalizability of these findings to other populations. In summary, future studies integrating multi-ethnic cohorts, exposome data, and functional validation are needed to clarify causal mechanisms linking EDC exposure to migraine. Given the widespread exposure to EDCs and the hormonal basis of migraine [55, 56], further investigation of EDC-migraine interactions have important implications for understanding disease biology and informing risk assessment and prevention strategies.

## Conclusions

This study provides integrative genetic and multi-omics evidence linking EDC-related molecular traits to migraine susceptibility. These findings suggest that EDC-related genes are associated with migraine and establish a framework for prioritizing candidate exposures and molecular targets for future mechanistic and therapeutic investigations.

## Supplementary Information

Below is the link to the electronic supplementary material.

Supplementary Material 1

## Data Availability

The eQTL summary data were obtained from the eQTLGen Consortium (https://www.eqtlgen.org/) and the GTEx V8 database (https://gtexportal.org/home/). The sQTL blood data are available from the eQTLGen Consortium. The mQTL blood data were derived from a meta-analysis of the Brisbane Systems Genetics Study and the Lothian Birth Cohorts, and can be accessed through the corresponding study resources. The pQTL blood data were sourced from the large-scale proteomic study by Pietzner et al., with access available via the study’s designated data repository. The migraine GWAS dataset is publicly available from OpenGWAS (ukb-b-16868 and finn-b-MIGRAINE_ TRIPTAN, https://opengwas.org/). Raw gene expression data for migraine can be downloaded from the ArrayExpress database under accession number E-MTAB-13397 (https://www.ebi.ac.uk/arrayexpress/). All datasets used in this study are publicly accessible as specified, enabling further investigation of the genetic mechanisms underlying migraine. Graphics was created with BioRender.com.

## Abbreviations

EDC: Endocrine-disrupting chemicals
SMR: Summary data-based Mendelian randomization
HEIDI: Heterogeneity in dependent instruments
TWAS: Transcriptome-wide association study
QTL: Quantitative trait locus
GWAS: Genome-wide association study
TPP: Triphenyl phosphate
B[a]P: Benzo(a)pyrene
PNKP: Polynucleotide kinase 3'-phosphatase
PRDM16: PR/SET domain 16
MEF2D: Myocyte enhancer factor 2D
HTRA1: HtrA serine peptidase 1
B9D2: B9 domain containing 2
LRCH1: leucine rich repeats and calponin homology domain containing 1
OSBPL10: Oxysterol binding protein like 10
STEAP3: Six-transmembrane epithelial antigen of prostate 3
